# Some Influences of the Darkened Streets

**Published:** 1915-01-16

**Authors:** 


### The Hospital

The Workers' Newspaper of Administrative Medicine and Institutional Life, Administration, National Insurance and Health.

No. 1491, Vol. LVII. SATURDAY, JANUARY 16, 1915. PRICE ONE PENNY.

### SOME INFLUENCES OF THE DARKENED STREETS.

It is a trite remark that there are two sides to every question, though it is not an unhappy faculty which on some issues permits us only to see one of them. Just now we cultivate the application of this faculty to the cause for which the nation is fighting, and we have to try to apply it also to the consequences which the struggle entails. Amidst these there are, of course, losses which no philosophy can redeem. Lessened incomes, heavier taxes, and darkened streets are slighter evils, and though we grumble at them, we may find their influence not altogether an unhappy one. The victim of a street accident, or the pedestrian who misses his way, may think the certainty of unlit lamps worse than the risk of a Zeppelin bomb. But these are prejudiced witnesses, and the rest of us may cultivate some benefit from the limitations which are imposed on us.

It is certain that in some respects the social life of the Metropolis has been modified by the defective street lighting now dictated by the authorities. Journeys and visits are less readily undertaken, and this necessarily means an increased cultivation of life at home. An evening spent at what is usually called "a place of amusement" becomes a serious matter when the return journey means an uncertain voyage through gloomy and half-lighted streets and suburbs. This may equal some educational and artistic loss to the individual, but it has the advantage that it preserves him for several hours from an atmosphere which is not distinguished either by purity or by successful ventilation. The risk of "chill" is the popular dread, and whatever be the mechanism by which this is accomplished, there is less chance of it at home than in the entrances and exits of a crowded hall.

A more continued domestic life means almost inevitably an earlier hour for retiring to bed. Perhaps the cynics will say such a step is an effort to escape boredom, but whatever the motive, the practice, we think, is entirely in the direction of health. Late hours involve, at least very often, sitting in stuffy, ill-ventilated rooms, with an atmosphere rich in the fumes of tobacco; and when there is nothing very definite to do there arises the health-sapping conviction that all is vanity and vexation of mind. Perhaps this disturbs the blood-pressure. No one quite knows; but it certainly disturbs the temper and does not help either the individual or the household. Again, is there not something substantial in the old-fashioned view that sleep before midnight carries a special and particular merit? There may be a simple and obvious explanation of this creed, but we are satisfied that, in any event, the practice it proposes is beneficial. To take a single illustration: it is widely known by medical practitioners that anæmic girls may long be treated with want of success until they are compelled to cultivate the habit of early to bed. And though strong and vigorous young men may with apparent impunity continue to hear the chimes at midnight, middle age announces itself by some dulness of the faculties on the days following such experiences. That different individuals need different amounts of sleep is certain, and it is perhaps true that sleep, like other habits, may be carried to excess; yet once the elasticity of youth is gone most of us are better and more fit when we avoid late hours. So far as the discipline of the war through the influence of the darkened streets leads to this result the lesson will have its advantages, and some of our readers, we fancy, are ready to give personal testimony to this effect. A European war is a costly method of learning wisdom, but as the war is here in any case, we may as well get out of it what good is possible.

Another example of the far-reaching force of the war is seen in the influence it is exercising on the playing habits of our poorer children. Those who are familiar with the crowded districts of South and East London know that for many school children the street is the only playground. Further, in many places, as the mothers are engaged in factory work, the home is often closed to the youngsters until seven or eight o'clock in the evening. Now, under the conditions of street lighting at present in operation, the dangers of the street as a playground have become substantially increased. The added risk does not deter the children from their games, but their escapes must sometimes be by the skin of their teeth; and there are other than physical dangers in the darkness. From all this has arisen a greater pressure on the "play centres" so honourably associated with the name of Mrs. Mary A. Ward. It seems that applications for admission to these centres have greatly increased; and the mothers themselves bring their children and beg that they be taken in. The work done by these centres is most excellent. They contribute to the happiness and, just now particularly, to the safety of children whose lot at the best is not a very fortunate one. If the war in darkening our streets directs the attention of those who can give help to these very desirable ends, its influence will not have been wholly evil.

